# DNA copy number variations in children with vesicoureteral reflux and urinary tract infections

**DOI:** 10.1371/journal.pone.0220617

**Published:** 2019-08-12

**Authors:** Dong Liang, Kirk M. McHugh, Pat D. Brophy, Nader Shaikh, J. Robert Manak, Peter Andrews, Inessa Hakker, Zihua Wang, Andrew L. Schwaderer, David S. Hains

**Affiliations:** 1 Department of Pediatrics, Indiana University, Indianapolis, IN, United States of America; 2 Department of Medical and Molecular Genetics, Indiana University School of Medicine, Indianapolis, IN, United States of America; 3 Division of Anatomy, The Ohio State University, Columbus, OH, United States of America; 4 Department of Pediatrics, University of Rochester Medical Center, Rochester, NY, United States of America; 5 Department of Pediatrics, University of Pittsburgh, Pittsburgh, PA, United States of America; 6 Departments of Biology and Pediatrics, University of Iowa, Iowa City, IA, United States of America; 7 Cold Spring Harbor Laboratory, Cold Spring Harbor, NY, United States of America; 8 Riley Hospital for Children at Indiana University Health, Indianapolis, IN, United States of America; German Cancer Research Center (DKFZ), GERMANY

## Abstract

Vesicoureteral reflux (VUR) is a complex, heritable disorder. Genome-wide linkage analyses of families affected by VUR have revealed multiple genomic loci linked to VUR. These loci normally harbor a number of genes whose biologically functional variant is yet to be identified. DNA copy number variations (CNVs) have not been extensively studied at high resolution in VUR patients. In this study, we performed array comparative genomic hybridization (aCGH) on a cohort of patients with a history of both VUR and urinary tract infection (UTI) with the objective of identifying genetic variations responsible for VUR and/or UTI susceptibility. UTI/VUR-associated CNVs were identified by aCGH results from the 192 Randomized Intervention for Children With Vesicoureteral Reflux (RIVUR) patients compared to 683 controls. Rare, large CNVs that are likely pathogenic and lead to VUR development were identified using stringent analysis criteria. Because UTI is a common affliction with multiple risk factors, we utilized standard analysis to identify potential disease-modifying CNVs that can contribute to UTI risk. Gene ontology analysis identified that CNVs in innate immunity and development genes were enriched in RIVUR patients. CNVs affecting innate immune genes may contribute to UTI susceptibility in VUR patients and may provide the first step in assisting clinical medicine in determining adverse outcome risk in children with VUR.

## Introduction

Primary vesiocoureteral reflux (VUR), characterized by retrograde flow of urine from the bladder to the ureter, affects 1–2% of children[1]. VUR is associated with renal scarring risk, hypertension and chronic kidney disease secondary to recurrent urinary tract infections (UTIs), which only affect a subset of children [2, 3]. Multiple VUR management recommendations exist but are largely limited to imaging and do not offer insight into treatment strategies to maximize benefit while limiting risks. Clearly, an increased understanding of factors that predispose some VUR patients to complications is needed.

The Randomized Intervention for Children with Vesicoureteral Reflux (RIVUR) trial randomized children with a history of UTI and VUR to daily antibiotic prophylaxis (AP) or placebo and followed them for two years to monitor for UTI recurrence risk[4]. Daily AP reduced the risk of UTI in children with VUR by almost 50% [4].

VUR is a heritable condition. One third of siblings of children with VUR will also have the condition [2]. Additionally, conditions associated with VUR complications such as recurrent UTI and pyelonephritis also present in familial patterns suggesting that VUR and recurrent UTIs may involve multiple overlapping genetic loci [5]. Although past studies have identified genetic variations responsible for a small subset of mostly syndromic VUR patients, the majority genetic variations responsible for primary VUR remains unknown [1, 6, 7]. Therefore, VUR is a complex genetic condition. Additionally, genetic analysis has not included traits linked to VUR sequelae such as UTI susceptibility.

Copy number variations (CNV) represent structural genomic variations comprising chromosomal segments that deviate from classic Mendelian diploid gene numbers [8]. Hundreds of CNVs have been identified that cause differential gene expression. Due to their ability to exert gene dosage-dependent effects, CNVs have been linked to structural kidney disease and infectious diseases [9–11].

Because VUR severity and UTI burden are variable, we hypothesized that CNVs in pathways critical to innate immunity and renal development will segregate with distinct clinical outcomes and phenotypes in VUR patients. We used high-resolution array comparative genomic hybridization (aCGH)-based genetic analysis to determine the CNV profile of children from The Randomized Intervention for Children with Vesicoureteral Reflux (RIVUR) trial with VUR and UTI and compared to race/gender-matched controls.

## Results

### CNV calls and filtering results

**Fig 1:**
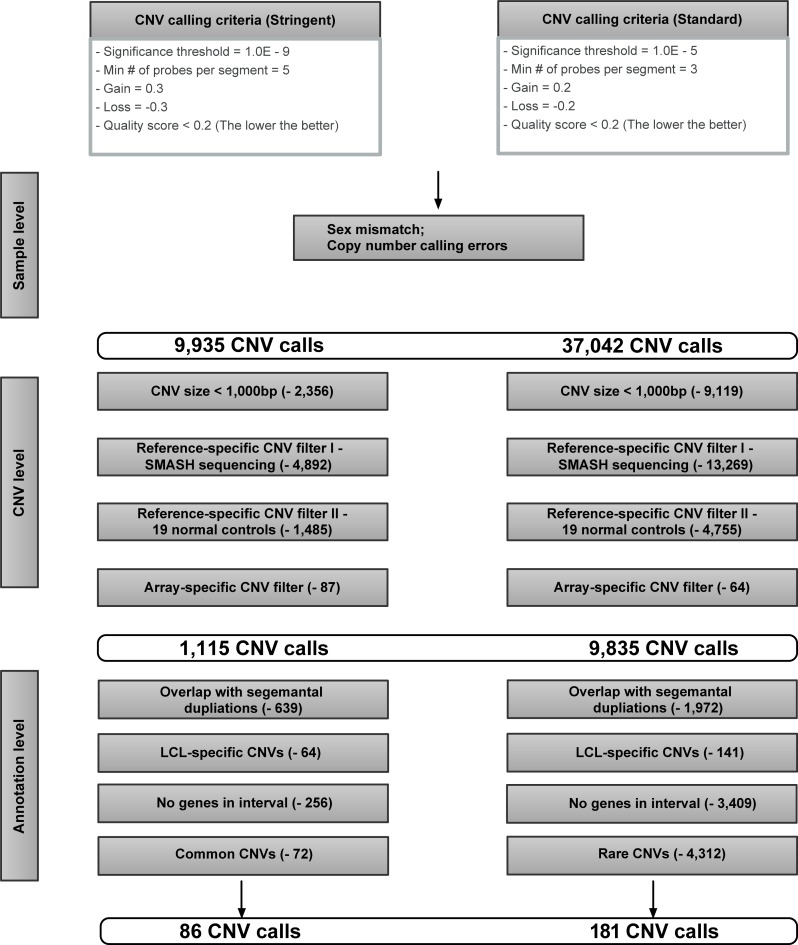
CNV calling, analysis, filtering methodology and results. In addition to GC waviness correction and log2 ratio filtering, we applied a rigid quality control pipeline at sample, CNV and annotation levels to identify disease-associated CNV candidates. In particular, the results of the SMASH sequencing of the two reference genomes (Reference-specific CNV filter I) as well as the CNV frequencies among the two control cohorts whose genomes were interrogated using the same Nimblegen platform (Reference-specific CNV filter II) were used to determine the reference genome-specific CNVs. Furthermore, the array-specific CNV filter directly compared the probe maps of both the Nimblegen HD2 2.1M and Agilent 1M CHG arrays, and selected regions that contain the common probe coverage area for further CNV comparisons. These procedures ensure that the quality of our analysis at overall genome level is not systematically interfered with the potential bias that would occur with the different reference samples and CGH detection platforms used in this study.

We performed high-resolution genome-wide aCGH arrays on 192 RIVUR patients and compared the results to 683 gender/race/ethnicity-matched controls **(S1 Fig)**. To identify VUR and UTI associated CNVs, we assessed the CNV frequencies in genomic DNA using standard and stringent CNV calling criteria and results filtering **(Fig 1)**. Because VUR is a relatively common disorder (1–2% of population) and UTIs are even more common, we utilized two different analysis methods in this study. Standard methodology resulted in a comprehensive candidate list, but difficult to interpret clinical relevance. We further utilized stringent analysis to look for strongly significant “true positive” candidates at the expense of eliminating some candidates that may be clinically relevant.

### The majority of the rare CNVs in VUR and UTI were less than 10kb and were gain events.

**Fig 2:**
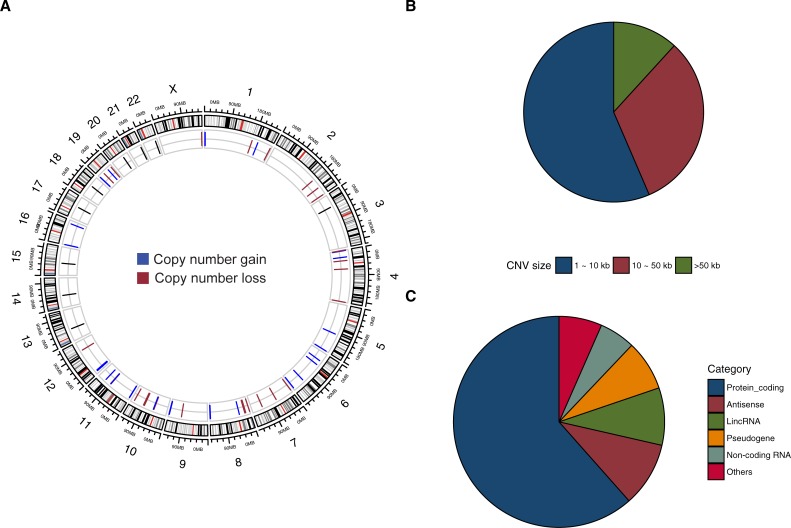
Rare candidate CNVs in RIVUR patients identified using stringent analysis criteria. (A) The genomic location of the rare candidate CNVs in RIVUR patients is shown in the circular plot. The rare (frequency < = 1% of controls), significant CNVs were displayed in the inner circle. Only regions of copy number gain (blue) and copy number loss (red) with significant adjusted P values are plotted. (B) The identified CNVs were categorized based on the CNV size range into difference size bins, and the percentage of CNVs in each bin are shown in the pie chart. (C) Pie chart reflects type of genic region spanned by CNVs. A large proportion of the identified CNVs intersect genic regions that encode for proteins.

Our stringent analysis resulted in identification of 85 significantly altered chromosomal regions with differential CNV frequencies **(Fig 1).** The sizes of these rare CNVs detected by aCGH have a median CNV size of 7,770 bp **(S2A Fig)**. Among all the CNVs, 47.1% represent copy number loss **(S2B Fig)**. The chromosomal locations and size distribution of the rare CNVs were analyzed and shown in **Fig 2A and 2B**. Additionally, among all the gene-affecting CNVs, 61.8% were found to intersect genomic regions that encode protein sequences. The remaining non-coding region encompassed genetic components including antisense (9.9%), long intergenic non-coding RNA (lincRNA) (8.8%), pseudogene (7.7%), non-coding RNA (5.5%), etc **(Fig 2C and S1 Table).**

Within the significantly altered CNVs (adjusted P-value < 0.05, based on a FDR adjustment for multiple testing), 85 candidate CNVs affecting 141 genes **(S2 Table)** passed our quality control filtering under the stringent analysis criteria. **S3 Fig** shows the top 20 most significantly altered rare CNVs and their affected genes in RIVUR patients versus controls. Among all the differentially altered CNVs, the chromosomal locus–chr12:11,215,990–11,217,836, is the top ranked CNV region harboring a 1.84-kb DNA segment of copy number gain. We detected this CNV in 33 (17.1%) of the RIVUR patients as compared with 0 controls, showing a significant enrichment (adjusted *P* = 4.84 x 10^−21^). This region harbors Proline Rich Protein HaeIII Subfamily 1 (*PRH1*) and two readthrough genes, *PRH1-PRR4* and *PRH1-TAS2R14*.

### Biological pathway analysis of CNVs demonstrate alterations in gene pathways critical in lower urinary tract development.

To gain insight into the potential biological consequence of rare CNVs, we performed GO pathway analysis of the candidate CNVs **(S2 Table).** The GO analysis revealed multiple enrichments for developmental pathways. Of note, “regulation of Wnt signaling pathway” (*P* = 0.0008) and “developmental growth involved in morphogenesis” (*P* = 0.018) are represented in this list. These pathways contain statistically significantly altered genes that have known roles in kidney development and/or known causes of VUR. The complete GO pathway analysis is presented in **S3 Table**. Additionally, we filtered our results against RefSeq for terms consistent with kidney and lower urinary tract development (see complete methods). CNVs with potential lower urinary tract development relevance involved multiple genes including Fibroblast Growth Factor Receptor 3 (*FGFR3*) and Tenascin XB (*TNXB*) (**Table 1**and **S2 Table**) [12–14]. Finally, we compared our results with previous linkage analysis studies in VUR, and we identified six genes that overlap with genes identified by linkage analysis in other VUR cohorts (**Table 2**) [6, 15–23].

**Table 1:** Summary of selected, rare candidate genes with known roles in normal kidney development.

Symbol			Name		Event	Freq_case (%)	Freq_ctl (%)
TNXB			tenascin XB		CN Gain	10.4	0.1
ZNF595			zinc finger protein 595		CN Loss	2.1	0
DVL1			dishevelled segment polarity protein 1		CN Gain	1.6	0
MMP23B			matrix metallopeptidase 23B		CN Gain	1.6	0
PCSK4			proprotein convertase subtilisin/kexin type 4		CN Gain	1.6	0
SKI			SKI proto-oncogene		CN Gain	1.6	0
SLC34A3	solute	carrier	family 34 (type II sodium/phosphate cotransporter), member	3	CN Gain	1.6	0
FGFR3			fibroblast growth factor receptor 3		CN Gain	1.6	0

**Table 2:** Rare candidate genes in VUR susceptibitly loci identified by prior genetic analysis studies.

Chromosome	Cytoband	Population	Study	Affected genes
1	1q23.2-1q25.2	Ireland	Kelly, 2007(17)	RABGAP1L
1	1q25-1q41	Europe	Sanna-Cherchi, 2013(23)	CFH; RABGAP1L
2	2q37.2-2q37.3	Ireland	Kelly, 2007(17)	LINC01237
6	6q24.1-6q27	Ireland	Kelly, 2007(17)	AFDN
6	6q27	Slovenia	Cordell, 2010(21)	AFDN
10	10q25.2-10q26.3	Ireland	Kelly, 2007(17)	*DMBT1
10	10q26.13	Ireland	Darlow, 2014(22)	*DMBT1
11	11q14.1	UK/Slovenia	Cordell, 2010(21)	DLG2

*: Genes with > 5% CNV frequency in the RIVUR cohort.

### Common CNVs in innate immune pathways may confer UTI susceptibility.

**Fig 3:**
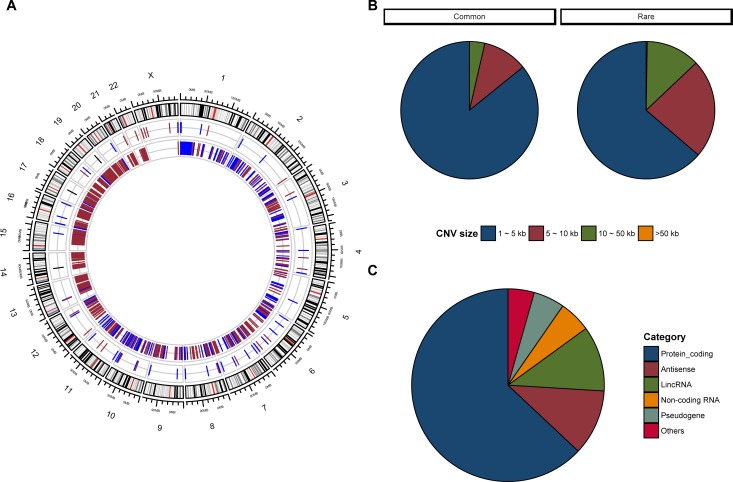
Disease-associated CNVs in RIVUR patients identified using standard analysis criteria. (A) The genomic location of disease-associated CNVs in RIVUR patients is shown in the circular plot. The common and rare (frequency < = 1% of controls) CNVs were displayed in the middle and inner circles, respectively. Only regions of copy number gain (blue) and copy number loss (red) with significant adjusted *P* values are plotted. (B) The disease-associated, common and rare CNVs were categorized based on the CNV size range into difference size bins, and the percentage of CNVs in each bin are shown in the pie charts. (C) Pie chart reflects type of genic region spanned by CNVs.

Unlike classic genetic studies, we also performed an analysis on “common” CNVs. We justify this approach as UTIs are a common condition and multifactorial, thus multiple genes likely contribute to innate defenses of the kidney and urinary tract. Our standard analysis resulted in identification of multiple significantly altered chromosomal regions with differential CNV frequencies **(Fig 1, Fig 3**and **S4 Table).** Among all the disease-associated CNVs, ninety-two are common disease-associated CNVs that may contribute to the spectrum of UTI burden (**S5 Table**). Thirty-four percent of these common CNVs were copy number loss events. Common CNVs were smaller and less likely to be losses compared to rare CNVs **(S4 Fig)**. **S5 Fig** shows the top 20 most significantly altered comme and rare CNVs and their affected genes in RIVUR patients versus controls.

### CNVs in genes critical to innate immunity and epithelial structure/function are significantly different in children with VUR/UTIs compared to controls.

GO pathway analysis of common CNVs significantly associated with VUR/UTI based on adjusted P values are presented in **S5 Table.** We have combined several similar pathways from **S6 Table** to highlight statistically significant unique processes in **Table 3.** We identified pathways critical in collecting duct development, innate immune response and receptor signaling. Furthermore, CNVs within enriched pathways included genes that encode antimicrobial peptides such as (Human alpha defensin 1–3, (*DEFA1A3*) and bacterial agglutins such as Deleted in Malignant Brain Tumor 1 (*DMBT1*).

**Table 3:** Summary of pathways involving common disease-associated CNVs.

Pathway (gene ontology)	Overlap	*P*-value	Genes
neuromuscular junction development (GO:0007528)^a^	3/34	5.08E-04	DVL1;AGRN;PDZRN3
negative regulation of glial cell proliferation (GO:0060253)^b^	2/9	7.38E-04	NOTCH1;SOX11
spindle assembly involved in mitosis (GO:0090307)^c^	2/11	1.12E-03	OFD1;ARHGEF10
collecting duct development (GO:0072044)	2/11	1.12E-03	NOTCH1;DACT2
response to muramyl dipeptide (GO:0032495)	2/12	1.34E-03	NOTCH1;CARD9
extracellular matrix assembly (GO:0085029)	2/15	2.11E-03	THSD4;GPM6B
antibacterial humoral response (GO:0019731)^d^	2/26	6.33E-03	DMBT1;DEFA1
response to fungus (GO:0009620)	2/35	1.13E-02	CARD9;DEFA1
receptor clustering (GO:0043113)	2/36	1.19E-02	DVL1;AGRN
hematopoietic progenitor cell differentiation (GO:0002244)	3/106	1.29E-02	COL24A1;DACT2;DOCK1
cilium morphogenesis (GO:0060271)	2/50	2.22E-02	OFD1;NOTCH1

^a^same genes as synapse organization (GO:0050808); P-value 0.0146155

^b^same genes with CNVs as cardiac ventricle formation (GO:0003211), cardiac chamber formation (GO:0003207), regula- tion of glial cell proliferation (GO:0060251), morphogenesis of an epithelial sheet (GO:0002011), ventricular septum mor- phogenesis (GO:0060412), mesenchymal cell development (GO:0014031) positive regulation of BMP signaling pathway (GO:0030513), stem cell development (GO:0048864), negative regulation of gliogenesis (GO:0014014), cardiac septum morphogenesis (GO:0060411), skeletal muscle cell differentiation (GO:0035914); P-values 0.0011205–0.019785

^c^same genes as microtubule cytoskeleton organization involved in mitosis (GO:1902850, mitotic spindle organization (GO:0007052), spindle assembly (GO:0051225); P-values 0.0018374–0.0138875

^d^same genes as antimicrobial humoral response (GO:0019730); P-value 0.0073198

By searching RefSeq, we queried gene function of statistically significant CNV-associated genes for known roles in innate immunity (**S7 Table**)(Comprehensive Methods for terms). We identified CNVs with potential innate immune functions involved genes that involve cytokines or chemokines such as Interleukin 11 (*IL11*), pattern recognition receptors such as Toll-Like Receptor 9 (*TLR*9), and antimicrobial peptides such as azurocidin 1 (*AZU1*) [24, 25] **(S7 Table)**.

## Discussion

Previous studies have found VUR-associated genetic mutations in pedigrees of mostly syndromic, patients, but these genetic variations have not been found in the vast majority of patients with primary, nonsyndromic VUR [1, 7]. Multiple reasons exist for the limited identification of gene defects responsible for VUR and include focus on SNPs as opposed to other types of genetic mutations such as CNVs. Also because VUR without recurrent UTIs may not lead to morbidities, it may be more clinically important to identify genetic factors that result in UTI risk.

The Nimblegen HD2 and Agilent 1M feature arrays cover polymorphic regions often omitted from conventional SNP arrays [26]. Prior VUR genetic studies often relied on elimination of “common” genetic variations as the first step in data analysis. Because UTIs are common, our analysis includes genetic variations for both common variations and rare variations. The genes affected by common CNVs may influence UTI risk in the appropriate clinical context such as bowel/bladder dysfunction and/or VUR. In future studies of newly diagnosed VUR patients, these genes can be investigated to predict UTI risk and/or antibiotic efficacy. In fact, one of our genes identified from our cohort was validated to show these exact associations, *DEFA1A3*. We have previously demonstrated renal collecting duct expression of *DEFA1A3*, positively correlated *DEFA1A3* copy number with expression levels [27]. We also demonstrated that RIVUR patients have low *DEFA1A3* copy number compared to age, sex and race/ethnicity matched controls with no UTI history [27]. Furthermore, *DEFA1A3* copy number also was associated with antibiotic prophylaxis efficacy suggesting synergy with antibiotics. If we had limited our analysis to only include the stringent CNV calls and filters, we would not have identified this very important locus, which we have independently validated as a complementary study to this data presented [27].

Our stringent analysis identified 85 rare CNVs. While members of these families as well as upstream or downstream signaling effectors have been studied in depth, many genes presented are novel. Of note, cytoband 12p13.2 is the top ranked chromosomal region that harbors multiple significantly altered CNVs **(S3 Fig)**. Interestingly, the genes affected by these CNVs encompass a number of genes including *PRH1*, *PRH1-PRR4* and *PRH1-TAS2R14*. The role of these genes have not been investigated in kidney development. *PRH1* is a parotid-specific gene with importance in innate defenses in the mouth and genetic variations are associated with risk for dental caries [28]. *PRH1-TAS2R14* produces a fusion protein. *TAS2R14* has been shown to inhibit mast cell degranulation [29]. Mast cells have been shown in various UTI models to be critical in bacterial clearance [30]. Because this is a gain event, conceivably one of these genes could be critical in innate defenses of the kidney and urinary tract and further investigation is warranted.

GO analysis was performed to determine biological processes enriched for CNV associated genes in RIVUR patients. Many of the identified biological processes involved innate immunity. Because enrollment in the RIVUR study required 1–2 documented UTIs, these innate immune gene CNVs may represent a UTI risk factor in RIVUR patients compared to controls. Key innate immune CNV genes identified in our analysis included *DEFA1A3*, *DMBT1*, and *CARD9*. *CARD9* is a critical adaptor protein for multiple innate immune processes and Toll-like receptor signaling [31]. *DMBT1* has been demonstrated to agglutinate Gram-positive and Gram-negative bacteria [32].

Prior research has focused on the developmental cause of VUR, and a number of murine models of VUR exist. These models indicate that the induction site of the ureteric bud during early kidney development gives rise to an abnormally tunneled ureter in the bladder and a subsequent faulty ureterovesical junction that leads to VUR [33]. Because gene dosage effects have led to ureteric bud induction infidelities in murine models, CNVs serve as the ideal structural genetic variation that could lead to the spectrum of VUR severity that we observe in children. Additionally, more than half of our CNVs were copy number gain events. Because most animal studies or *in vitro* studies involve loss of function, the biological relevance of a copy number gain in many candidate genes is unknown. Studies using validatory *in vivo* vertebrate models to upregulate these genes is needed to determine relevance. Of note, Tenascin XB (*TNXB*) has previously been associated with VUR [14]. *TNXB* single nucleotide polymorphism was postulated to be a gain-of-function event. Interestingly, in RIVUR, CNVs in *TNXB* predicted as coding sequence variants were gain events, which correlates with the previously known genetic events.

In addition to *TNXB1*, we identified additional CNVs affecting genes that have been implicated in lower urinary tract development. SRY-Box11(*SOX11*) has been reported to play a key regulatory role in renal development and its disruption has been implicated in causing congenital anomalies of the kidney and urinary tract [34]. Fibroblast Growth Factor (*FGF*) Receptor 3 is expressed during normal kidney development [33, 35]. NOTCH signaling is implicated in Alagille syndrome which has a high rate of VUR, and we identified CNVs in *NOTCH1* [36]. Dishevelled Segment Polarity Protein 1 (DVL1) is critical in WNT signaling during kidney development [37]. Additionally, Dishevelled Binding Antagonist of Beta Catenin 2 (*DACT2*) was identified in our studied and has a known role in collecting duct development [38]. Finally, we have also identified genes and non-coding lncRNA and miRNAs that are novel, and their roles in lower urinary tract development have yet to be investigated.

This study provides new insights into VUR/UTI pathophysiology, however we do acknowledge some limitations. While our aCGH provides very high-resolution data, we used 2 different aCGH platforms in a portion of the RIVUR patients due to commercial availability. We have run a subset of the same samples on both platforms and confirmed that the Agilent array does not detect additional CNVs found in the Nimblegen array. We did, however, demonstrate that less CNVs are detected, which we postulate is secondary to the lower resolution of the array. Additionally, the SFARI control cohort used a different reference genome. To account for these analysis factors, we filtered results for both aCGH array type and reference genome. We have also used SMASH sequencing to establish if any reference genome CNVs exist and filtered our results accordingly. Because the immortalization process to create our DNA source can result in differential genomic structural variations, we have filtered our results to exclude regions implicated [39]. Finally, our control population is a group of unaffected mothers from a cohort of probands with autism spectrum. While there are no known associations with VUR and autism, we do acknowledge that this comparison group is not perfect. Because VUR resolves over time and the rates of *in utero* VUR are unknown, we essentially cannot find a “pure” comparison cohort that we are certain does not have a history of VUR even if a voiding cystourethrogram was performed for reasons other than urinary tract abnormalities or UTI.

Development of a genetic panel to identify patients at risk for sequalae such as recurrent UTI and subsequent renal scarring would help “low risk” children avoid unneeded antibiotics and radiation exposure as well as select “high risk” patients for more aggressive treatment. Because the initial step of using genetic profiles is to improve care of children with VUR, we have identified several novel findings relevant to VUR/UTI pathophysiology including: 1) VUR patients with a UTI history are more likely to have CNVs involving innate immune genes compared to controls, 2) VUR patients with a UTI history are more likely to have CNVs that involve ureteric bud/collecting duct development pathways than controls, and 3) aCGH identified overlap between VUR loci identified in this study and prior linkage studies. Because the clinical course of children with vesicoureteral reflux is what is critical in clinical practice, determining those at risk for UTI is essential in managing children who are diagnosed with vesicoureteral reflux. Results from our study will serve as the foundation to inform medical decision-making and the first step in personalized medicine for this patient population.

## Methods

For complete methods, please refer to the supporting information file (**S1 File**). The workflow of the overall experimental design is outlined in **S1 Fig**. Approval on human subjects was obtained by Nationwide Children’s Hospital Institutional Review Board (IRB) protocols IRB07-00383 and IRB10-00319 and the University of Tennessee Health Science Center IRB protocol 14-03325-XP. All clinical investigations were conducted according to the principles expressed in the Declaration of Helsinki.

### Subjects

RIVUR cohort (cases): Randomized Intervention for Children with Vesicoureteral Reflux (RIVUR) Study (ClinicalTrials.gov Identifier NCT00405704): For complete study design and outcome data, please refer to previously published materials and the supporting information file (**S1 File**). [4, 40]. Briefly, this clinical trial enrolled 607 children aged 1–71 months with documented VUR by voiding cystourethrogram grades I-IV and have 1 but no more than 2 documented UTI’s.

SFARI cohort (controls): To use samples of unrelated individuals, we selected unaffected mothers from families of Simons Foundation Autism Research Initiative (SFARI) cohort for case-control comparison [41]. The SFARI is a scientific initiative within the Simons Foundation that focuses on autism spectrum disorders (https://sfari.org). We collected results from patients from the Simons-Simplex Collection whose DNA was analyzed with the Nimblegen HD2 2.1 million probe microarray platform (3). The complex trait of idiopathic autism is not related to any developmental or innate immunity phenotypes of VUR.

Local control cohort (controls): A total of 19, ethnicity/race-, sex- matched healthy controls with no prior history of VUR or UTI served as the control group in this study. These 19 control subjects’ genomes were interrogated on the Nimblegen HD2 platform with the same reference genome as the RIVUR subjects. Additionally, these 19 ethnicity/race-, sex- matched local healthy controls were also used for filtering purposes. Specifically, the SFARI cohort and our local controls were interrogated on the same Nimblegen HD2 platform but with different reference genomes. In order to control for potential reference-specific CNVs, we compared the CNV frequency difference between the 19 normal controls (which had the same reference as the RIVUR samples) and the SFARI cohort. Whenever a 10% threshold of difference is identified, the involved CNV was labeled as dubious positive and excluded from all the downstream analysis as it was most likely attributed to a reference genome CNV.

### RIVUR vs Control comparison

For case-control comparison, the case group consists of 192 non-Hispanic, Caucasian females from the RIVUR cohort. The control group is composed of 19 healthy controls as well as 664 unrelated sex and race/ethnicity matched samples from SFARI cohort.

### Short multiply aggregated sequence homologies (SMASH) sequencing

We identified the copy number variants (CNVs) of reference DNA samples by SMASH [42]. A detailed description of SMASH can be found in the supporting information file (**S1 File**).

### aCGH arrays

For our DNA quantification as well as high-resolution aCGH methodology, please refer to our previous work and the supporting information file (**S1 File**) [43].

### Genome-wide CNV calls

The aCGH data was processed using Nexus 8 Copy Number software (Biodiscovery Inc, El Segundo, CA). For specific copy calling parameters, please refer to the supporting information file (**S1 File**).

### Quality control and association analysis

We peformed filtering to standardize for array platform (Nimblegen vs. Agilent) and reference DNA. In order to correct for the possible genomic changes in lymphoblastic cell line, we specifically filtered out genomic regions that harbor putative LCL-sepcific changes before further analysis. (lymphoblastic cell line derived [39] vs. primary DNA). For a flow chart of the CNVs calling criteria, analyses, filtering criteria as well as the data to demonstrate the effectiveness of filtering, please refer to **Fig 1**and the supporting information file (**S1 File**).

### CNV annotation and enrichment analysis

The CNVs were classified into common or rare (< 1% in controls) CNVs based on their frequency in control group. The gene ontology enrichment pathway analysis were performed on the selected candidate genes. The information of clinical significance and most severe effect for each candidate CNV were retrieved from the ClinVar and the Ensemble Variant Effect Predictor databases, respectively.

### Overlap analysis with previous VUR genetic studies

The rare candidate CNV-affected genes were mapped to the previously identified VUR susceptibility loci [6, 15–23].

## Supporting information

S1 Fig Flowchart of experimental design. (EPS)

S2 Fig Characteristics of rare candidate CNVs identified using stringent criteria. (A) shows the cumulative distribution of CNVs by size. (B) reflects the distribution of copy number (CN) gains versus CN losses of rare candidate CNVs identified using stringent analysis criteria.
(EPS)

S3 Fig The top 20 rare candidate CNVs and their affected genes identified using stringent criteria. The frequency differences (bar) and the level of significance transformed as—log10(*P*) are plotted. * indicates the adjusted—log10(*P*) values. The dashed black line shows where the significant threshold (*P* = 0.05) lies.
(EPS)

S4 Fig Characteristics of common versus rare disease-associated CNVs. (A) A higher total percentage of common CNVs are smaller compared to rare CNVs using standard analysis criteria.(B) A majority of common CNVs are gains, while the majority of rare CNVs are loss events.
(EPS)

S5 Fig **The top 20 common (A) and rare (B) disease-associated CNVs and their affected genes identified using standard criteria.** The frequency differences (bar) and the level of signifi- cance transformed as—log10(*P*) are plotted. * indicates the adjusted—log10(*P*) values. The dashed black line shows where the significant threshold (*P* = 0.05) lies.
(EPS)

S6 Fig Example of segmentation and whole genome array plots. Panel A and B show that a copy number loss or gain is detected within the *DMBT1* or *WWOX* gene locus, respectively. Panel C shows the entire genome with probes, a moving average line and colored chromosomes linked end to end.
(EPS)

S1 Table Type of genic regions spanned by rare candidate CNVs identified using stringent analysis criteria. (DOCX)

S2 Table Summary of rare candidate CNVs and their affected genes identified using stringent analysis criteria. (XLSX)

S3 Table Gene ontology enrichment analysis of rare candidate CNV-affected genes identified using stringent analysis criteria. (XLSX)

S4 Table Type of genic regions spanned by disease-associated CNVs identified using standard analysis criteria. (DOCX)

S5 Table Summary of disease-associated candidate genes and CNVs identified using standard analysis criteria. (XLSX)

S6 Table Gene ontology enrichment analysis of common candidate CNV-affected genes identified using standard analysis criteria. (XLSX)

S7 Table Summary of selected disease-associated candidate genes with known roles in innate immunity. (DOCX)

S1 File Comprehensive/supplemental materials and methods. (DOC)
