# Supplementary material for: DNA copy number variations in children with vesicoureteral reflux and urinary tract infections
Source: PLoS One. 2019 Aug 12;14(8):e0220617. doi: 10.1371/journal.pone.0220617 (PMC6690579; doi:10.1371/journal.pone.0220617)
Supplement: S1 Table — (DOCX) [file pone.0220617.s007.docx]

**S1 Table. Type of genic regions spanned by rare candidate CNVs identified using stringent analysis criteria**

| Category | N | Percent (%) |
| --- | --- | --- |
| protein_coding | 56 | 61.5 |
| antisense | 9 | 9.9 |
| lincRNA | 8 | 9.9 |
| pseudogene | 7 | 7.7 |
| Non-coding RNA | 5 | 5.5 |
| processed_transcript | 3 | 3.3 |
| sense_intronic | 2 | 2.2 |
| sense_overlapping | 1 | 2.2 |
