# Supplementary material for: DNA copy number variations in children with vesicoureteral reflux and urinary tract infections
Source: PLoS One. 2019 Aug 12;14(8):e0220617. doi: 10.1371/journal.pone.0220617 (PMC6690579; doi:10.1371/journal.pone.0220617)
Supplement: S4 Table — (DOCX) [file pone.0220617.s010.docx]

| **Category** | **Common CNV** | **Rare CNV** |
| --- | --- | --- |
|  | **N Percent (%)** | **N Percent (%)** |
| protein_coding | 67 74.4 | 1410 69.5 |
| antisense | 10 11.1 | 239 11.8 |
| lincRNA | 4 4.4 | 141 6.9 |
| processed_transcript | 3 3.3 | 33 1.6 |
| Non-coding RNA | 2 2.2 | 111 5.5 |
| pseudogene | 2 2.2 | 61 3 |
| Inactivated immunoglobulin gene | 1 1.1 | 0 0 |
| polymorphic_pseudogene | 1 1.1 | 1 0 |
| sense_intronic | 0 0 | 24 1.2 |
| sense_overlapping | 0 0 | 9 0.4 |
| 3prime_overlapping_ncrna | 0 0 | 1 0 |

**S4 Table. Type of genic regions spanned by disease-associated CNVs identified using standard analysis criteria**
