# Supplementary material for: DNA copy number variations in children with vesicoureteral reflux and urinary tract infections
Source: PLoS One. 2019 Aug 12;14(8):e0220617. doi: 10.1371/journal.pone.0220617 (PMC6690579; doi:10.1371/journal.pone.0220617)
Supplement: S7 Table — (DOCX) [file pone.0220617.s013.docx]

| **Symbol** | **Name** | **Event** | **Freq_case (%)** | **Freq_ctl (%)** | **-log10(*q*)** |
| --- | --- | --- | --- | --- | --- |
| CFH | complement factor H | CN Gain | 13.5 | 0.0 | 16.8 |
| STAB1 | stabilin 1 | CN Gain | 13.0 | 0.1 | 14.8 |
| IL11 | interleukin 11 | CN Loss | 8.3 | 0.0 | 9.8 |
| THNSL2 | threonine synthase-like 2 | CN Gain | 4.7 | 0.0 | 5.3 |
| TOR2A | torsin family 2, member A | CN Gain | 4.7 | 0.0 | 5.3 |
| ITIH1 | inter-alpha-trypsin inhibitor heavy chain 1 | CN Gain | 4.7 | 0.1 | 4.5 |
| C8G | complement component 8, gamma polypeptide | CN Gain | 5.2 | 0.3 | 4.4 |
| CSF1 | colony stimulating factor 1 (macrophage) | CN Gain | 2.6 | 0.0 | 2.8 |
| LGMN | legumain | CN Loss | 2.6 | 0.0 | 2.7 |
| NLRP8 | NLR family, pyrin domain containing 8 | CN Loss | 2.6 | 0.0 | 2.7 |
| AZU1 | azurocidin 1 | CN Loss | 2.1 | 0.0 | 2.1 |
| ELANE | elastase, neutrophil expressed | CN Loss | 2.1 | 0.0 | 2.1 |
| LRG1 | leucine-rich alpha-2-glycoprotein 1 | CN Loss | 2.1 | 0.0 | 2.1 |
| IRF4 | interferon regulatory factor 4 | CN Gain | 1.6 | 0.0 | 1.6 |
| LTC4S | leukotriene C4 synthase | CN Gain | 1.6 | 0.0 | 1.6 |
| MAPK11 | mitogen-activated protein kinase 11 | CN Gain | 2.1 | 0.1 | 1.6 |
| TLR9 | toll-like receptor 9 | CN Gain | 1.6 | 0.0 | 1.6 |
| PPARA | peroxisome proliferator-activated receptor alpha | CN Loss | 1.6 | 0.0 | 1.5 |

**S7 Table. Summary of selected disease-associated candidate genes with known roles in innate immunity**
