# Supplementary material for: DNA copy number variations in children with vesicoureteral reflux and urinary tract infections
Source: PLoS One. 2019 Aug 12;14(8):e0220617. doi: 10.1371/journal.pone.0220617 (PMC6690579; doi:10.1371/journal.pone.0220617)
Supplement: S1 File — (DOC) [file pone.0220617.s014.doc]

**SUPPLEMENTARY MATERIAL**

LIST OF SUPPLEMENTARY MATERIALS

**S1 Fig.** Flowchart of experimental design

**S2 Fig.** Characteristics of rare candidate CNVs identified using stringent criteria

**S3 Fig.** The top 20 rare candidate CNVs and their affected genes identified using stringent criteria

**S4 Fig.** Characteristics of common versus rare disease-associated CNVs

**S5 Fig.** The top 20 common (A) and rare (B) disease-associated CNVs and their affected genes identified using standard criteria

**S6 Fig.** Example of segmentation and whole genome array plots

**S1 Table.** Type of genic regions spanned by rare candidate CNVs identified using stringent analysis criteria

**S2 Table.** Summary of rare candidate CNVs and their affected genes identified using stringent analysis criteria

**S3 Table.** Gene ontology enrichment analysis of rare candidate CNV-affected genes identified using stringent analysis criteria

**S4 Table.** Type of genic regions spanned by disease-associated CNVs identified using standard analysis criteria

**S5 Table.** Summary of disease-associated candidate genes and CNVs identified using standard analysis criteria

**S6 Table.** Gene ontology enrichment analysis of common candidate CNV-affected genes identified using standard analysis criteria

**S7 Table.** Summary of selected disease-associated candidate genes with known roles in innate immunity

COMPREHENSIVE/SUPPLEMENTAL MATERIALS AND METHODS

The workflow of the overall experimental design is outlined in Fig. S1. Approval on human subjects was obtained by Nationwide Children’s Hospital Institutional Review Board (IRB) protocols IRB07-00383 and IRB10-00319 and the University of Tennessee Health Science Center IRB protocol 14-03325-XP. All clinical investigations were conducted according to the principles expressed in the Declaration of Helsinki.

**Subjects**

RIVUR cohort (cases): Randomized Intervention for Children with Vesicoureteral Reflux (RIVUR) Study (ClinicalTrials.gov Identifier NCT00405704): For complete study design and outcome data, please refer to previously published materials [1, 2]. Briefly, in this multi-center, randomized, placebo-controlled clinical trial, 607 children aged 1-71 months had documented VUR by voiding cystourethrogram grades I-IV and have one but no more than 2 documented UTI’s. Children were randomized to daily antibiotic prophylaxis or placebo and followed for 2 years. Study participants were monitored for urinary tract infections via phone interview every 2 months, clinic visits every 6 months, and DMSA renal scans to monitor for scarring at enrollment, 12, and 24 months. Patients that developed new scars on DMSA scans during the study were characterized as “new scars.” Patient DNA was obtained and immortalized lymphoblastic cell lines established for 455 participants.

SFARI cohort (controls): To use samples of unrelated individuals, we selected unaffected mothers from families of Simons Foundation Autism Research Initiative (SFARI) cohort for case-control comparison [3]. The SFARI is a scientific initiative within the Simons Foundation that focuses on autism spectrum disorders ([https://sfari.org](https://sfari.org/)). We collected results from patients from the Simons-Simplex Collection whose DNA was analyzed with the Nimblegen HD2 2.1 million probe microarray platform (3). The complex trait of idiopathic autism is not related to any developmental or innate immunity phenotypes of VUR.

Local control cohort (controls): A total of 19, ethnicity/race-, sex- matched healthy controls with no prior history of VUR or UTI served as the control group in this study. These control subjects’ genomes were interrogated on the Nimblegen HD2 platform with the same reference genome as the RIVUR subjects to control for potential reference-specific CNVs.

**RIVUR vs Control comparison**

For case-control comparison, the case group consists of 192 non-Hispanic, Caucasian females from the RIVUR cohort. The control group is composed of 19 healthy controls as well as 664 unrelated sex and race/ethnicity matched samples from SFARI cohort of autism patients. The raw Nimblegen aCGH data of SFARI cohort was obtained from NCBI’s Gene Expression Omnibus through GEO Series accession number GSE23682 (<http://www.ncbi.nlm.nih.gov/geo/query/acc.cgi?acc=GSE23682>).

**DNA preparation**

Briefly, the saliva samples from the local control group were collected using Oragene DNA collection kits (DNA Genotek, Inc., Ontario, Canada), and the genomic DNAs were extracted with the Gentra Puregene kits per manufacturer’s recommendations (Qiagen, Valencia, CA). This saliva collection method yields a mean of 13 and 85 micrograms of DNA from children and adults, respectively.

**Short multiply aggregated sequence homologies (SMASH) sequencing**

We identified the copy number variants (CNVs) of reference DNA samples by SMASH, a next-generation sequencing-based method for CNV analysis [4]. Briefly, short DNA fragments (~40 bp) were obtained from 400 ng of genomic DNA by dsDNA fragmentase. After end-repairing the DNA fragments, we ligated them together and enriched 300-700 bp DNA molecules. We then ligated these chimeric DNA molecules with sequencing adapters and carried out the final PCR by index primers for a few cycles. For mapping SMASH data, we applied a time-efficient and memory-intensive algorithm to conservatively divide the chimeric reads into uniquely mappable fragments. In the same manner as read maps, the fragment maps were utilized to detect CNVs using read-depth of coverage. In this study, we performed the CNV analysis at 1m bin resolution, with the average size of 3 kb/bin and about 40 mappable fragments per bin.

**aCGH arrays**

The patient and control genomic DNA samples were quantified and assessed for quality using a NanoDrop spectrophotometer (Thermo Scientific; Wilmington, DE) and agarose gel electrophoresis. For NimbleGen HD2 2.1M feature aCGH and quality control parameters, please refer to our previous work [5].

For Agilent 1M feature arrays, 0.5 µg of genomic DNA from an experimental and a healthy male reference sample were labeled and amplified with Cy3- or Cy5-coupled random nonamers (Agilent), respectively, according to manufacturer’s instructions (CGH Enzymatic Labeling Kit Protocol v.7.3). Sample or control DNA was added with water to a Random Primer Buffer (Agilent), heated to 98°C for 10 min, and held in ice water for 5 min. Next, 19 µL of Labeling mix (water, fluorescent nonamers, dNTPs, Klenow 3’-5’ enzyme) was added to tubes which were then placed at 37°C for 2 hrs followed by 65°C for 10 minutes. Next, 1X TE Buffer pH8 was added to the reaction tubes and the solution was placed into an Agilent Purification column. The reaction underwent two washes with TE Buffer and was eluted into a fresh tube. TE Buffer was then added to the sample to bring it to a volume of 80.5 µL and its specific activity was measured using Nanodrop. The experimental and control samples were then combined along with hybridization buffer (Agilent) and pipetted onto a microarray chip using a gasket slide and placed at 42°C for 40-48 hrs in a hybridization oven (Agilent). After this incubation period, microarray chips were washed with two sequential wash buffers (Agilent) and scanned at 2 µm (pixel size) with Agilent SureScan Microarray Scanner, at two wavelengths: 635 nm and 532nm. Scanned images were aligned with a design file and their fluorescence was measured with Agilent Cytogenomics software.

**Genome-wide CNV calls**

The aCGH data was processed using Nexus 8 Copy Number software (Biodiscovery Inc, El Segundo, CA). A systemic correction process was performed to correct for the possible data waviness. Copy number segmentation was then performed using the Fast Adaptive States Segment Techniques 2 (FASST2) segmentation algorithm implemented in the Nexus Copy Number software. We used both standard and stringent CNV calling criteria to identify potential disease-modifying CNVs and likely pathogenic, rare CNVs, respectively. Specifically, two sets of CNV calling parameters were used to identify likely pathogenic (stringent) and potential disease-modifying (standard) CNVs. Briefly, to obtain high-confidence CNV calls, a very stringent significance threshold of 1 x 10^-9^  (1 x 10^-5^) *P*-value; a minimum number of five (three) contiguous probes per segment, as well as >= 0.3 (0.2) log2 ratio threshold of gain or loss were applied in the stringent (standard) version of CNVs calling. The CNVs are defined in this study as variable segments of DNA of >= 1kb in length and same copy number events.

**Quality control and association analysis**

We used the following quality control pipeline to ensure the integrity of our data and analysis**.** Briefly, at sample level, first we discarded subjects with sex mismatch or CNV calling errors (e.g. excessive CNV calls across all X chromosome) by visual inspection of frequency plots of individual samples. We also eliminated subjects that had quality control scores > 0.2 based on probe-to-probe variance of log ratios calculated by the Nexus software.

At CNV level, we excluded the CNVs with DNA segments of < 1kb in length. To account for possible reference genome CNVs that could affect analyses, we performed the whole genome SMASH sequencing of DNAs of the two reference genomes used in the CGH experiments. When sequencing results differed in copy numbers between the two genomes, those CNVs were eliminated from analyses (Reference-specific CNV filter I). Furthermore, we also excluded CNVs whose frequency in 19 normal controls were more than 10% different relative to that in SFARI cohort. Specifically, for those significantly altered CNV loci, we further compared the CNV frequency difference between the 19 normal controls and disease controls of SFARI cohort. Whenever a 10% threshold of difference is identified, the involved CNV was labeled as dubious positive and excluded from all the downstream analysis as it was most likely attributed to a reference genome CNV (Reference-specific CNV filter II). These procedures greatly eliminate the possibility that the observed association is driven by the difference in reference genomes used. Additionally, in order to ensure the validity of CNV comparisons, we also removed the array-specific CNVs by performing a probe map-based, probe coverage analysis that only keeps regions with coverage of at least 3 probes for both Nimblegen HD2 2.1M and Agilent 1M CGH arrays (Array-specific CNV filter).

Finally, at annotation level, we filtered out the CNVs with reciprocal overlap with segmental duplications or lymphoblastic cell line (LCL) -specific CNVs[6] and those without any genes in the interval. To identify rare, likely pathogenic CNVs or common, potential disease-modifying CNVs, we also excluded CNVs with => 1% or < 1 % incidence in controls, respectively. For a complete flow chart of the CNVs calling criteria, analyses and filtering criteria as well as the data to demonstrate the effectiveness of filtering, please refer to **Fig. 1**.

The association analysis was performed using a Fisher’s exact test to access the CNV frequency difference between cases and controls based on random chance within each CNV locus (NEXUS 8 Copy Number software). For RIVUR vs Control comparison, both unadjusted and adjusted P values correcting for multiple testing by performing false discovery rate correction are reported. A difference with adjusted P value of 0.05 or less was considered statistically significant. The candidate gene list was generated by choosing the genes affected by significantly altered CNVs. For each gene locus, the significant CNV with the lowest adjusted *P* and the coordinates of all other significant CNVs within that gene were reported.

**CNV annotation and enrichment analysis**

The CNVs were classified into common or rare (< 1% in controls) CNVs based on their frequency in control group. The significant CNVs identified in RIVUR vs Control comparison were annotated across the GENCODE database (Release 19 mapped to GRCh37, http://www.gencodegenes.org/releases/19.html) to determine the coverage of genomic features. The gene ontology enrichment and KEGG pathway analysis were performed on the selected candidate genes identified in the aCGH analysis between RIVUR patients versus controls (adjusted P value < 0.05) using the enrichment tools (version 2015) in the EnrichR platform ([http://amp.pharm.mssm.edu/Enrichr/](http://david.ncifcrf.gov/)) [7]. The clinical significance information was annotated for candidate CNVs that overlap with the reported human genetic variations in the ClinVar database (<https://www.ncbi.nlm.nih.gov/clinvar/>). To assess the likelihood of pathogenicity, the most severe effects of the candidate CNVs were also predicted through the online Ensemble Variant Effect Predictor tool (https://useast.ensembl.org/info/docs/tools/vep/index.html).

**Selected candidates**

To further determine biological relevance of our list of candidate genes, we cross-reference our candidate list against RefSeq for terms 'kidney', 'renal', 'bacteria', 'antimicrobial', 'bacterial’, 'inflammatory', ‘inflammation’, ‘infection’, 'vesicoureteral', 'ureteric', 'fibrosis', 'scarring', 'congenital', 'development’, 'collecting duct' and 'bladder'.

**Overlap analysis with previous VUR genetic studies**

The genomic coordinates of cytoband location (hg19, GRCh37) were downloaded from UCSC Genome Annotation Database (http://hgdownload.cse.ucsc.edu/goldenpath/hg19/database/cytoBand.txt.gz). The likely pathogenic candidate genes were mapped to the previously identified VUR susceptibility loci with nonparametric linkage scores (or logarithm of odds scores) greater than 2 for VUR or 1.5 for urinary tract malformations [8-17]. The overlapped genes with > 5 % CNV frequencies in the RIVUR cohort are denoted by asterisks.

**Reference**

1. Carpenter MA, Hoberman A, Mattoo TK, Mathews R, Keren R, Chesney RW, et al. The RIVUR trial: profile and baseline clinical associations of children with vesicoureteral reflux. Pediatrics. 2013;132(1):e34-45. doi: 10.1542/peds.2012-2301. PubMed PMID: 23753091; PubMed Central PMCID: PMC3691529.

2. Investigators RT, Hoberman A, Greenfield SP, Mattoo TK, Keren R, Mathews R, et al. Antimicrobial prophylaxis for children with vesicoureteral reflux. N Engl J Med. 2014;370(25):2367-76. doi: 10.1056/NEJMoa1401811. PubMed PMID: 24795142; PubMed Central PMCID: PMCPMC4137319.

3. Levy D, Ronemus M, Yamrom B, Lee YH, Leotta A, Kendall J, et al. Rare de novo and transmitted copy-number variation in autistic spectrum disorders. Neuron. 2011;70(5):886-97. doi: 10.1016/j.neuron.2011.05.015. PubMed PMID: 21658582.

4. Wang Z, Andrews P, Kendall J, Ma B, Hakker I, Rodgers L, et al. SMASH, a fragmentation and sequencing method for genomic copy number analysis. Genome Res. 2016;26(6):844-51. doi: 10.1101/gr.201491.115. PubMed PMID: 27197213; PubMed Central PMCID: PMCPMC4889966.

5. Brophy PD, Alasti F, Darbro BW, Clarke J, Nishimura C, Cobb B, et al. Genome-wide copy number variation analysis of a Branchio-oto-renal syndrome cohort identifies a recombination hotspot and implicates new candidate genes. Hum Genet. 2013;132(12):1339-50. doi: 10.1007/s00439-013-1338-8. PubMed PMID: 23851940; PubMed Central PMCID: PMCPMC3830662.

6. Shirley MD, Baugher JD, Stevens EL, Tang Z, Gerry N, Beiswanger CM, et al. Chromosomal variation in lymphoblastoid cell lines. Hum Mutat. 2012;33(7):1075-86. doi: 10.1002/humu.22062. PubMed PMID: 22374857; PubMed Central PMCID: PMCPMC3370055.

7. Zarrei M, MacDonald JR, Merico D, Scherer SW. A copy number variation map of the human genome. Nat Rev Genet. 2015;16(3):172-83. doi: 10.1038/nrg3871. PubMed PMID: 25645873.

8. Feather SA, Malcolm S, Woolf AS, Wright V, Blaydon D, Reid CJ, et al. Primary, nonsyndromic vesicoureteric reflux and its nephropathy is genetically heterogeneous, with a locus on chromosome 1. Am J Hum Genet. 2000;66(4):1420-5. doi: 10.1086/302864. PubMed PMID: 10739767; PubMed Central PMCID: PMCPMC1288208.

9. Sanna-Cherchi S, Reese A, Hensle T, Caridi G, Izzi C, Kim YY, et al. Familial vesicoureteral reflux: testing replication of linkage in seven new multigenerational kindreds. J Am Soc Nephrol. 2005;16(6):1781-7. doi: 10.1681/ASN.2004121034. PubMed PMID: 15829711.

10. Kelly H, Molony CM, Darlow JM, Pirker ME, Yoneda A, Green AJ, et al. A genome-wide scan for genes involved in primary vesicoureteric reflux. J Med Genet. 2007;44(11):710-7. doi: 10.1136/jmg.2007.051086. PubMed PMID: 17660461; PubMed Central PMCID: PMCPMC2752186.

11. Conte ML, Bertoli-Avella AM, de Graaf BM, Punzo F, Lama G, La Manna A, et al. A genome search for primary vesicoureteral reflux shows further evidence for genetic heterogeneity. Pediatr Nephrol. 2008;23(4):587-95. doi: 10.1007/s00467-007-0675-z. PubMed PMID: 18197425; PubMed Central PMCID: PMCPMC2259258.

12. Weng PL, Sanna-Cherchi S, Hensle T, Shapiro E, Werzberger A, Caridi G, et al. A recessive gene for primary vesicoureteral reflux maps to chromosome 12p11-q13. J Am Soc Nephrol. 2009;20(7):1633-40. doi: 10.1681/ASN.2008111199. PubMed PMID: 19443636; PubMed Central PMCID: PMCPMC2709685.

13. Briggs CE, Guo CY, Schoettler C, Rosoklija I, Silva A, Bauer SB, et al. A genome scan in affected sib-pairs with familial vesicoureteral reflux identifies a locus on chromosome 5. Eur J Hum Genet. 2010;18(2):245-50. doi: 10.1038/ejhg.2009.142. PubMed PMID: 19690587; PubMed Central PMCID: PMCPMC2987194.

14. Cordell HJ, Darlay R, Charoen P, Stewart A, Gullett AM, Lambert HJ, et al. Whole-genome linkage and association scan in primary, nonsyndromic vesicoureteric reflux. J Am Soc Nephrol. 2010;21(1):113-23. doi: 10.1681/ASN.2009060624. PubMed PMID: 19959718; PubMed Central PMCID: PMCPMC2799286.

15. Puri P, Gosemann JH, Darlow J, Barton DE. Genetics of vesicoureteral reflux. Nat Rev Urol. 2011;8(10):539-52. doi: 10.1038/nrurol.2011.113. PubMed PMID: 21862976.

16. Sanna-Cherchi S, Sampogna RV, Papeta N, Burgess KE, Nees SN, Perry BJ, et al. Mutations in DSTYK and dominant urinary tract malformations. N Engl J Med. 2013;369(7):621-9. doi: 10.1056/NEJMoa1214479. PubMed PMID: 23862974; PubMed Central PMCID: PMCPMC3846391.

17. Darlow JM, Dobson MG, Darlay R, Molony CM, Hunziker M, Green AJ, et al. A new genome scan for primary nonsyndromic vesicoureteric reflux emphasizes high genetic heterogeneity and shows linkage and association with various genes already implicated in urinary tract development. Mol Genet Genomic Med. 2014;2(1):7-29. doi: 10.1002/mgg3.22. PubMed PMID: 24498626; PubMed Central PMCID: PMCPMC3907909.
